# Anti-Obesity Effect of Diphlorethohydroxycarmalol Isolated from Brown Alga *Ishige okamurae* in High-Fat Diet-Induced Obese Mice

**DOI:** 10.3390/md17110637

**Published:** 2019-11-10

**Authors:** Yuling Ding, Lei Wang, SeungTae Im, Ouibo Hwang, Hyun-Soo Kim, Min-Cheol Kang, Seung-Hong Lee

**Affiliations:** 1 Department of Pharmaceutical Engineering, Soonchunhyang University, Asan 31538, Korea; dingyuling@naver.com (Y.D.); lama1010@naver.com (S.I.); ouiboing123@gmail.com (O.H.); 2 Department of Marine Life Sciences, Jeju National University, Jeju Self-Governing Province 63243, Korea; comeonleiwang@163.com; 3 Marine Science Institute, Jeju National University, Jeju Self-Governing Province 63333, Korea; 4 Department of Applied Research, National Marine Biodiversity Institute of Korea, Seochun 33662, Korea; gustn783@naver.com; 5 Research Group of Food Processing, Korea Food Research Institute, Wanju 55365, Korea

**Keywords:** high-fat diet mice, *Ishige okamurae*, diphlorethohydroxycarmalol (DPHC), anti-obesity effect

## Abstract

Diphlorethohydroxycarmalol (DPHC) is one of the most abundant bioactive compounds in *Ishige okamurae*. The previous study suggested that DPHC possesses strong in vitro anti-obesity activity in 3T3-L1 cells. However, the in vivo anti-obesity effect of DPHC has not been determined. The current study explored the effect of DPHC on high-fat diet (HFD)-induced obesity in C57BL/6J mice. The results indicated that oral administration of DPHC (25 and 50 mg/kg/day for six weeks) significantly and dose-dependently reduced HFD-induced adiposity and body weight gain. DPHC not only decreased the levels of triglyceride, low-density lipoprotein cholesterol, leptin, and aspartate transaminase but also increased the level of high-density lipoprotein cholesterol in the serum of HFD mice. In addition, DPHC significantly reduced hepatic lipid accumulation by reduction of expression levels of the critical enzymes for lipogenesis including SREBP-1c, FABP4, and FAS. Furthermore, DPHC remarkably reduced the adipocyte size, as well as decreased the expression levels of key adipogenic-specific proteins and lipogenic enzymes including PPARγ, C/EBPα, SREBP-1c, FABP4, and FAS, which regulate the lipid metabolism in the epididymal adipose tissue (EAT). Further studies demonstrated that DPHC significantly stimulated the phosphorylation of adenosine monophosphate-activated protein kinase (AMPK) and acetyl-CoA carboxylase (ACC) in both liver and EAT. These results demonstrated that DPHC effectively prevented HFD-induced obesity and suggested that DPHC could be used as a potential therapeutic agent for attenuating obesity and obesity-related diseases.

## 1. Introduction

Obesity is characterized as an excessive accumulation of body fat caused by an imbalance between energy intake and expenditure [1]. The incidences of obese and overweight people have increased worldwide and become a global public health issue. Obesity is associated with many metabolic disorders, including hypertension, type 2 diabetes, insulin resistance, and cardiovascular diseases [2,3]. In recent years, several synthetic drugs such as Xenical® (orlistat) and Reductil® (sibutramine) have been developed for the treatment of obesity through regulation of appetite, fat absorption, and fat oxidation [4,5]. However, the side-effects including insomnia, thirst, tension headaches, constipation, and steatorrhea of these drugs have been reported [6]. Owing to the advantages of natural compounds such as safety and efficiency, to find a therapeutic candidate from natural substances has taken more attention [7].

Seaweeds are abundant in natural bioactive compounds, such as proteins, sterols, polysaccharides, and polyphenols. These compounds possess various bioactivities including anti-inflammatory, anti-obesity, antioxidant, and anti-aging effects [8,9,10,11,12]. Some seaweeds have been used as food or medicinal ingredient for thousands of years in Asian countries, such as *Hizikia fusiforme*, *Sargassum fulvellum*, *Undaria pinnatifida*, and *Ishige okamurae* [8,10,13,14,15,16]. In recent years, increasing evidence support that seaweed consumption is associated with a lower risk of obesity as well as obesity-related complications [17,18]. Thus, several research studies have focused on investigating anti-obesity effects of algal-derived compounds [12,19,20]. 

**Figure 1:**
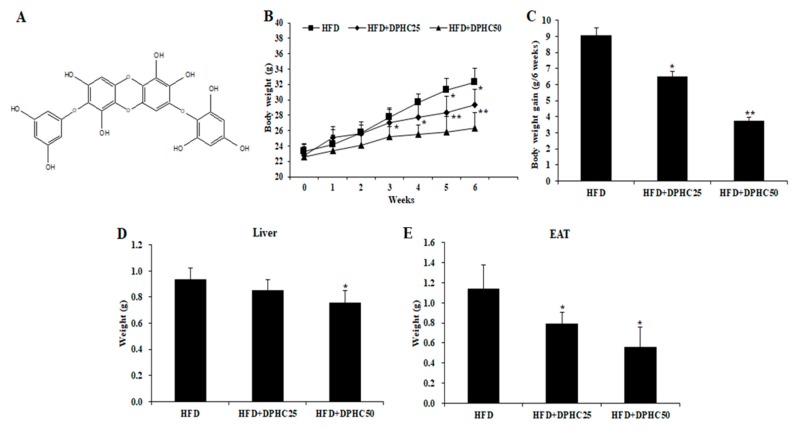
Diphlorethohydroxycarmalol (DPHC) reduces adiposity in high-fat diet (HFD)-induced obese mice. (**A**) Chemical structure of DPHC. The bodyweight (**B**) and the bodyweight grain (**C**) of the mice measured during six weeks HFD treatment or co-treated with DPHC. The weights of liver (**D**) and epididymal adipose tissue (EAT) (**E**) of HFD-induced mice. Data are expressed as mean ± SD (*n* = 8). Significant differences compared to HFD-induced mice were identified at * *p* < 0.05 and ** *p* < 0.01.

*I. okamurae* is an edible brown alga, which is abundant on the coast of Jeju Island, Korea. Diphlorethohydroxycarmalol (DPHC, Figure 1A) is one of the most abundant bioactive compounds of *I. okamurae*. Previous studies have reported that DPHC possesses strong anti-inflammatory, anti-diabetic, anti-oxidant, and anti-melanogenesis effects [21,22,23]. In addition, the in vitro anti-obesity effect of DPHC in 3T3-L1 cells has been investigated in the previous study [16]. The results indicated that DPHC effectively reduced the expression of adipocyte-specific proteins and the adipogenic enzymes in adipocytes, as well as inhibited the fat accumulation through activating acetyl-CoA carboxylase (ACC) and adenosine monophosphate-activated protein kinase (AMPK) in 3T3-L1 cells [16]. These results indicated that DPHC possesses strong anti-obesity effect and suggested the potential of DPHC in suppressing obesity. To further investigate the anti-obesity potential of DPHC, the effect of DPHC on high-fat diet (HFD)-induced obese mice has been evaluated in the present study.

## 2. Results

### 2.1. Effects of DPHC on Adiposity in HFD-Induced Obese Mice

In order to investigate the in vivo anti-obesity effect of DPHC, we fed mice with HFD or HFD supplemented with DPHC for six weeks and measured the body weights of mice weekly. As Figure 1B shows, the body weights of the mice fed with HFD supplemented with DPHC are significantly less than the mice fed with HFD only. The body weight gained in six weeks for the HFD fed mice was 9.1 g, and for the mice fed with HFD supplemented with 25 and 50 mg/kg body weight of DPHC were 6.5 and 3.8 g, respectively (Figure 1C). In addition, the mass of the liver and the epididymal adipose tissue (EAT) of mice were measured after six weeks. As the results show, the mice treated with DPHC had gained less liver (Figure 1D) and EAT (Figure 1E) mass than the mice fed with HFD only. These results indicated that HFD-stimulated adiposity in mice could be suppressed by DPHC treatment. 

### 2.2. Effects of DPHC on Serum Biochemical Parameters in HFD-Induced Obese Mice

The serum lipid metabolism-related biochemical parameters of the mice were measured using various enzyme-linked immunosorbent assay (ELISA) kits based on the manufacturer’s instructions. As the results in Table 1 show, the serum levels of low-density lipoprotein cholesterol (LDL-C), triglyceride (TG), and leptin were remarkably decreased; however, the high-density lipoprotein cholesterol (HDL-C) level was significantly increased in DPHC-treated mice. These results displayed that DPHC effectively regulated these biochemical parameters in HFD-induced obese mice. In addition, DPHC-treated mice had significantly lower aspartate transaminase (AST), which is indicative of liver damage, than the mice fed with HFD only. 

### 2.3. Effects of DPHC on Hepatic Lipid Accumulation and Regulated Lipid Metabolism-Related Proteins Expression in HFD-Induced Obese Mice

**Figure 2:**
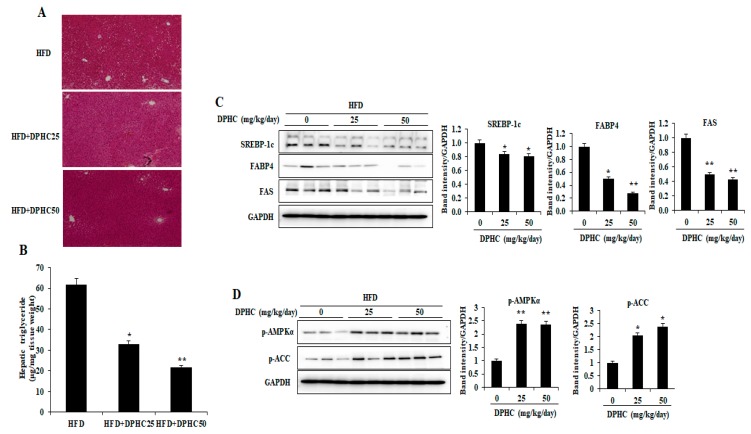
DPHC treatment inhibits hepatic lipid accumulation and regulates the expression of several key enzymes and specific proteins involved in lipid metabolism in HFD-induced obese mice. (**A**) The image of the representative HandE-stained liver section and (**B**) the relative hepatic TG levels. The expression levels of (**C**) sterol regulatory element-binding protein-1c (SREBP-1c), fatty acid synthase (FAS), and fatty acid-binding protein (FABP4) and (**D**) the phosphorylation levels of acetyl-CoA carboxylase (ACC) and adenosine monophosphate-activated protein kinase (AMPKα) were measured by western blot analysis. The data are expressed as mean ± SD (*n* = 8). Significant differences compared to HFD-induced mice were identified at * *p* < 0.05 and ** *p* < 0.01.

Adipocyte dysfunction in obesity influences lipid accumulation in the liver. We therefore examined the effects of DPHC on hepatic lipid accumulation using hematoxylin and eosin (HandE) staining. DPHC-treated mice had significantly reduced hepatic lipid accumulation compared to the HFD-fed mice (Figure 2A). This observation was clearly confirmed by measuring hepatic TG levels (Figure 2B). To investigate the mechanism of inhibited hepatic lipid accumulation by DPHC, expression levels of several key enzymes and specific proteins involved in lipid metabolism were measured by western blotting. As shown in Figure 2C, DPHC treatment significantly decreased expression levels of the critical enzymes for lipogenesis including fatty acid-binding protein (FABP4), sterol regulatory element-binding protein-1c (SREBP-1c), and fatty acid synthase (FAS). These results displayed that DPHC effectively reduced the hepatic lipid accumulation by inhibiting lipogenic enzymes in HFD-induced obese mice. Moreover, the phosphorylation levels of ACC and AMPKα, which are involved in fatty acid oxidation, were significantly increased with DPHC treatment (Figure 2D). Collectively, these results suggest that DPHC prevents hepatic steatosis through inhibition of lipid synthesis and up-regulation of fatty acid oxidation.

### 2.4. Effects of DPHC on Adipocyte Size and Expression Levels of Adipogenic-Specific Proteins in the Epididymal Adipose Tissue (EAT) of HFD-Induced Obese Mice

**Figure 3:**
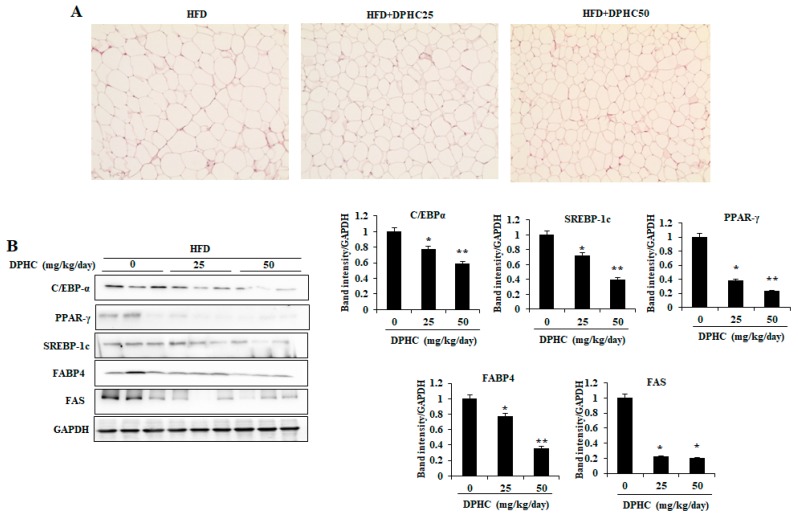
DPHC reduces the adipocyte size and regulates expression of the key proteins and enzymes related to lipid metabolism in EAT of HFD-induced obese mice. (**A**) The image of the representative HandE-stained EAT section. (**B**) The expression levels of SREBP-1c, FAS, FABP4, CCAAT/enhancement-binding protein-α (C/EBPα), and peroxisome proliferator-activated receptor-γ (PPAR-γ) were measured by western blot analysis. Data are expressed as mean ± SD (*n* = 8). Significant differences compared to HFD-induced mice were identified at * *p* < 0.05 and ** *p* < 0.01.

As Figure 3A shows, the mice treated with DPHC have smaller epididymal adipocyte size than the mice fed with HFD only. The expression levels of key adipogenic-specific proteins were investigated to elucidate the molecular mechanisms underlying the inhibitory effect of DPHC on adipogenesis. As shown in Figure 3B, DPHC treatment showed a significant decrease in the expression levels of adipogenic-specific proteins including CCAAT/enhancement-binding protein-α (C/EBPα), peroxisome proliferator-activated receptor-γ (PPAR-γ), and SREBP-1c in the EAT of HFD-induced obese mice. Moreover, DPHC also decreased the expression levels of FAS and FABP, which were related to lipid synthesis. These results suggest that DPHC suppresses the HFD-induced increase adipocyte size by reducing the expression of key adipogenic transcription factors and lipogenesis in EAT.

### 2.5. Effects of DPHC on Activation of AMPKα and Lipolytic Enzymes in the EAT of HFD-Induced Obese Mice

**Figure 4:**
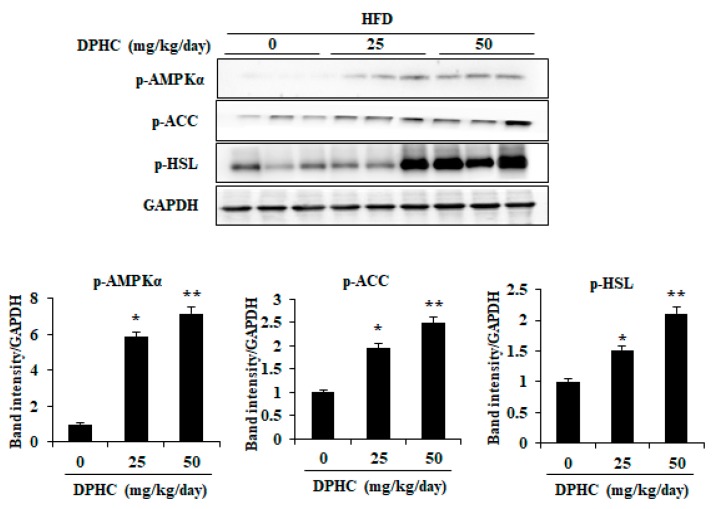
DPHC promotes activation of AMPKα, ACC, and hormone-sensitive lipase (HSL) in the EAT of HFD-induced obese mice. The phosphorylation levels were assessed by Western blot analysis. Data are expressed as mean ± SD (*n* = 8). Significant differences compared to HDF-induced mice were identified at * *p* < 0.05 and ** *p* < 0.01.

To determine whether other mechanisms might also be involved in the DPHC-induced reduction in adiposity, we investigated the effect of DPHC on the activation of AMPKα, ACC, and hormone-sensitive lipase (HSL) in EAT. As shown in Figure 4, DPHC-treated mice significantly increased phosphorylation of AMPKα and ACC compared with the only HFD-fed mice. Furthermore, DPHC treatment effectively promoted HSL phosphorylation in the EAT (Figure 4). Collectively, these results indicate that DPHC could inhibit lipid accumulation in EAT by activating AMPKα, ACC, and HSL on HFD-induced obesity. 

## 3. Discussion

Obesity is related to various chronic diseases, such as cardiovascular diseases, diabetes, nonalcoholic fatty liver disease, osteoarthritis, and some types of cancer [24,25]. It has become not only a public health problem but also a social issue in the 21st century. According to the report from Termmel et al. (2017), more than 2.1 billion individuals (nearly 30% of the global population) were obese or overweight, as well as 5% of deaths worldwide attributed to obesity in 2014 [26]. Thus, to investigate the mechanism of obesity and to discover the therapeutic agent against obesity has taken more attention.

Recently, a number of reports suggested that marine algae possess the potential in anti-obesity [27,28,29,30,31,32]. Kang et al. (2016) had investigated the anti-obesity effects of 27 kinds of seaweeds collected from Jeju Island, Korea [30]. The results indicated that the 70% ethanol extracts of these seaweeds effectively inhibited intracellular lipid accumulation in 3T3-L1 adipocytes [30]. In addition, Maeda et al. (2009) had reported on the anti-obesity and anti-diabetic effect of fucoxanthin isolated from the popular edible brown alga, *Undaria pinnatifida* [32]. The results suggested that fucoxanthin significantly improved lipid metabolism and insulin resistance in HFD-induced mice [32]. *I. okamurae* is a popular edible seaweed in Korea and it is rich in phenolic compounds. The in vitro anti-obesity effects of *I. okamurae* and its major bioactive compound, DPHC, had been investigated in the previous studies [16,30,33]. However, the in vivo anti-obesity effect of DPHC has not been investigated so far. Therefore, in the present study, we investigated the effect of DPHC on high-fat diet (HFD)-induced obesity in C57BL/6J mice.

The current results demonstrated that the bodyweight grain of HFD-induced mice was significantly and dose-dependently reduced by DPHC treatment (Figure 1C). The bodyweight gains of the HFD-induced mice were reduced from 9.1 g to 6.5 and 3.8 g (28.57 and 58.24% reduced) in the mice treated with 25 and 50 mg/kg body weight of DPHC, respectively (Figure 1C). Seo et al. (2018) had reported that the bodyweight gains of HFD-induced mice were reduced from 26.8 g to 18 g (32.84% reduced) by treatment with 300 mg/kg of ethanol extract of *I. okamurae* [34]. These results indicated that DPHC possesses around 10-folds anti-obesity effect than the ethanol extract of *I. okamurae* and suggested DPHC may be the main anti-obesity compound in *I. okamurae*. 

Lipid accumulation plays an important role in the development of obesity and is regulated by various adipogenic-specific proteins including SREBP-1c, PPARγ, and C/EBPα, as well as lipogenic enzymes such as FABP4 and FAS [16]. Thus, to inhibit lipid accumulation through regulation of these specific proteins and lipogenic enzymes may be a possible strategy against obesity. Various reports indicated that the phenolic compounds such as epigallocatechin-3-gallate isolated from green tea and dieckol isolated from brown seaweed, which reduced lipid accumulation in adipocytes through down-regulating the expressions of SREBP-1c, FABP4, FAS, PPARγ, and C/EBPα [35,36]. Our previous research suggested that DPHC significantly inhibited lipid accumulation by down-regulation of SREBP-1c, PPARγ, FABP4, C/EBPα, and FAS in vitro in 3T3-L1, which is through activation of AMPK and ACC [16]. However, the mechanism of the anti-obesity of DPHC still needs to be investigated via animal study. Therefore, in the present study, the expression levels of these key proteins and enzymes involved in lipid metabolism in the EAT of HFD-induced obese mice were measured. As Figure 3 shows, DPHC significantly reduced the expression levels of C/EBPα, PPARγ, SREBP-1c, FABP4, and FAS, which plays an important role in adipogenesis and lipogenesis, in EAT. These results demonstrate that the anti-obesity ability of DPHC is mediated by its inhibition of adipogenesis and lipogenesis in EAT. The current study identified the anti-obesity effect of DPHC in animal models, supporting previous in vitro studies. AMPK is a crucial upstream regulator of lipolysis and lipid oxidation in adipocytes and liver [37]. AMPK also plays an important role by directly phosphorylating the downstream target ACC. This phosphorylation inhibits its lipogenic enzyme activity and causes a subsequent decrease in lipid synthesis [38]. Therefore, the effects of natural products to prevent and treat obesity through AMPK activation have recently attracted researchers’ attention [39]. We found that DPHC treatment could significantly increase both AMPK and ACC phosphorylation in the EAT (Figure 4). In addition, HSL plays an important role in lipolysis. The phosphorylated HSL could lead to hydrolysis of diacylglycerol to liberate free fatty acids and monoacylglycerol [40]. As Figure 4 shows, DPHC significantly and dose-dependently induced HSL phosphorylation. Taken together, the present results suggest that DPHC might inhibit adipocyte differentiation and lipogenesis via AMPK activation.

Serum cholesterol levels are related to obesity and the high serum cholesterol level could lead to many metabolic diseases [41,42]. Seo et al. (2018) had reported that the ethanol extract of *I. okamurae*, which contains DPHC, effectively ameliorated the deleterious changes in blood metabolic parameters in HFD-induced obese mice [34]. It suggested the potential of DPHC in the regulation of the blood metabolic parameters in HFD-induced obese mice. Therefore, the levels of obesity-related blood metabolic parameters in the serum were evaluated. The results indicated that DPHC effectively regulated the obesity-related blood metabolic parameters in HFD-induced obese mice displayed in reducing TG, LDL-C, leptin, and AST levels, and increasing HDL-C levels (Table 1). These results indicated that DPHC effective ameliorates the deleterious changes in blood metabolic parameters and may prevent the metabolic diseases induced by the deleterious changes in blood metabolic parameters.

Lipid accumulation in the liver could lead to nonalcoholic fatty liver disease. The obesity-related lipid accumulation in the liver could develop to nonalcoholic steatohepatitis, which is a further development to fibrosis and chronic inflammation [43,44]. Thus, in this study, the effect of DPHC on hepatic lipid accumulation in HFD-induced mice and its mechanisms were investigated. The current results showed that DPHC significantly reduced hepatic lipid accumulation by down-regulation of the lipogenic-related enzymes including SREBP-1c, FAS, and FABP4, as well as up-regulation of the phosphorylated levels of AMPKα and ACC in the liver of HFD-induced obese mice (Figure 2). These results demonstrated that DPHC remarkably inhibits HFD-induced hepatic lipid accumulation by reducing the lipogenesis and enhancing lipolysis. In this way, DPHC could not only attenuate obesity but also prevents obesity-related hepatic diseases.

In summary, the present results suggested that DPHC possesses a strong effect against HFD-induced obesity through regulation of multi-pathways in vivo in obese mice. Combined with our previous studies, we can conclude that the phenolic compound isolated from edible seaweed *I. okamurae*, DPHC, possesses strong in vitro and in vivo anti-obesity activities and it could be used as a potential therapeutic agent for attenuating obesity and obesity-related diseases. 

## 4. Materials and Methods

### 4.1. Materials

The ELISA kits for measuring the levels of TG, HDL-C, and LDL-C were purchased from Abcam (Cambridge, UK). The kit for measuring leptin level was purchased from Invitrogen (Carlsbad, CA, USA). The kit for measuring the AST level was purchased from BioVision Inc (Milpitas, CA, USA). The antibodies against C/EBPα, PPAR-γ, FABP4, FAS, p-HSL, p-ACC, and p-AMPKα were purchased from Cell Signaling Technology (Bedford, MA, USA). The antibody against SREBP-1c was purchased from Santa Cruz Biotechnology (Santa Cruz, CA, USA). All other chemicals and reagents used in this study were analytical grade.

### 4.2. Extraction and Isolation of DPHC

*I. okamurae* was collected from the coast of Jeju Island, Korea, in April 2019. The protocol of extraction and isolation was described in our previous study [15]. Briefly, *I. okamurae* was extracted with 70% ethanol and the ethanol extract of *I. okamurae* (IO-E) was obtained. Then, IO-E was partitioned with ethyl acetate and the ethyl acetate fraction of IO-E (IO-E-EA) was obtained. IO-E-EA was subjected to silica gel and Sephadex-LH-20 column chromatography. Then, the DPHC (Figure 1A) was further purified by high-performance liquid chromatography and identified by comparing the NMR spectral data. 

### 4.3. Experimental Animals and Treatment

Male C57BL/6J mice (4 weeks of age) were purchased from Joongah Bio (Suwon, Korea). All mice were maintained in a temperature and humidity-controlled facilities on a 12 h light/dark cycle. The mice were allowed free access to commercial pellet chow and water. After 2 weeks acclimation, the mice were randomly divided into the 3 groups (*n* = 8): high-fat diet group (HFD, rodent diet with 45 kcal% fat), low-dose DPHC supplemented group (HFD + DPHC25), and high-dose DPHC supplemented group (HFD + DPHC50). DPHC was dissolved in distilled water (DW), and animals were orally administered with DPHC at a dose of 25 mg/kg body weight/day (HFD + DPHC25) or 50 mg/kg body weight/day (HFD + DPHC50) using oral feeding needles for 6 weeks. Mice in the HFD group were given an equal volume of DW. The body weights of mice were measured each week during the experimental period. Finally, the mice were anesthetized after withholding food for 12 h, and blood, liver and epididymal adipose tissue (EAT) were collected. The tissues were stored at −80 °C until analysis. The experimental protocol was approved by the Laboratory Animal Administration Committee of Soonchunhyang University and performed according to the University Guidelines for Animal Experimentation (Approval number: SCH19-0039).

### 4.4. Blood Parameter Analysis

All mice have fasted for 12 h before sacrifice. Blood was collected and the serum was separated by centrifuge (3000 × *g*, 10 min, and 4 °C). The TG, HDL-C, LDL-C, leptin, and AST levels in serum were determined using commercial assay ELISA kits according to the manufacturer’s instructions. 

### 4.5. Histological Analysis

The EAT and liver tissues were fixed with 10% formalin and embedded in paraffin. Sections were obtained and stained with HandE. The sections were photographed after dehydration by alcohol using a light microscope (Olympus D970, Olympus Optical Co., Tokyo, Japan). 

### 4.6. Western Blot Analysis

The EAT and liver tissues were homogenized and the protein concentrations were determined. The western blot analysis was performed following the protocol described by Ko et al. [35]. The signals were developed using an ECL Western Blotting Detection Kit and exposed to X-ray films.

### 4.7. Data and Statistical Analysis

Data are represented as means ± standard deviation (SD). The statistical analysis was performed using SPSS software. The values were evaluated by one-way analysis of variance (ANOVA) followed by post-hoc Duncan’s multiple range tests.

## Figures and Tables

**Table 1:** Effect of DPHC on the deleterious changes in blood metabolic parameters in HFD-induced obese mice.

Parameter	Group		
	HFD	HFD + DPHC25	HFD + DPHC50
TG (mg/dL)	137.88 ± 16.24	105.88 ± 4.09 *	86.73 ± 11.03 **
HDL-C (mg/dL)	50.49 ± 3.10	60.93 ± 7.12 *	72.71 ± 7.30 *
LDL-C (mg/dL)	22.24 ± 1.40	18.07 ± 1.15 *	16.82 ± 2.02 *
Leptin (ng/mL)	2.04 ± 0.59	1.48 ± 0.42	1.23 ± 0.37 *
AST (mU/mL)	47.11 ± 6.07	42.22 ± 3.74	41.02 ± 1.52 *

Data are expressed as mean ± SD (*n* = 8). Significant differences compared with HFD mice group were identified at * *p* < 0.05 and ** *p* < 0.01. HFD: mice fed with high-fat diet; HFD + DPHC25: mice fed with HFD and 25 mg/kg body weight/day of DPHC; HFF + DPHC50: mice fed with HFD and 50 mg/kg body weight/day of DPHC; TG: Triglyceride; HDL-C: low-density lipoprotein cholesterol; HDL-C: high-density lipoprotein cholesterol; AST: aspartate transaminase.
